# Molecular Markers of Antifungal Resistance: Potential Uses in Routine Practice and Future Perspectives

**DOI:** 10.3390/jof7030197

**Published:** 2021-03-09

**Authors:** Guillermo Garcia-Effron

**Affiliations:** 1 Laboratorio de Micología y Diagnóstico Molecular, Cátedra de Parasitología y Micología, Facultad de Bioquímica y Ciencias Biológicas, Universidad Nacional del Litoral, Santa Fe CP3000, Argentina; ggarcia@unl.edu.ar; Tel.: +54-9342-4575209 (ext. 135); 2 Consejo Nacional de Investigaciones Científicas y Tecnológicas, Santa Fe CP3000, Argentina

**Keywords:** antifungal resistance, molecular tools, intrinsic resistance, secondary resistance, Cyp51A, FKS, *Candida*, *Aspergillus*

## Abstract

Antifungal susceptibility testing (AST) has come to establish itself as a mandatory routine in clinical practice. At the same time, the mycological diagnosis seems to have headed in the direction of non-culture-based methodologies. The downside of these developments is that the strains that cause these infections are not able to be studied for their sensitivity to antifungals. Therefore, at present, the mycological diagnosis is correctly based on laboratory evidence, but the antifungal treatment is undergoing a growing tendency to revert back to being empirical, as it was in the last century. One of the explored options to circumvent these problems is to couple non-cultured based diagnostics with molecular-based detection of intrinsically resistant organisms and the identification of molecular mechanisms of resistance (secondary resistance). The aim of this work is to review the available molecular tools for antifungal resistance detection, their limitations, and their advantages. A comprehensive description of commercially available and in-house methods is included. In addition, gaps in the development of these molecular technologies are discussed.

## 1. Introduction

Antifungal susceptibility testing is an essential tool in different clinical scenarios. Standardized protocols (from the Clinical and Laboratory Standards Institute (CLSI)and from the European Committee on Antimicrobial Susceptibility Testing (EUCAST)) and commercially available methods (some of them automated) are able to detect resistant fungal strains, to guide antifungal therapies, and to offer reliable epidemiological data on antifungal resistance [1]. When these methodologies seemed to have come to establish themselves as a mandatory routine in any clinical microbiology laboratory, the mycological diagnosis seems to have headed in the direction of non-culture-based methodologies. These techniques, which include serological and molecular-based tools, improve mycological diagnosis in speed, sensitivity, and specificity. A proven invasive mycosis can be diagnosed by amplifying fungal DNA from a paraffin-embedded tissue [2] or by detecting a fungal antigen (e.g., *Cryptococcus* spp.) with a speed previously dreamed of [3]. Moreover, there are commercially available molecular-based methods able to detect fungal DNA with good sensitivity and speed [4]. The downside of these developments is that the strains that cause these infections are not available to study their sensitivity to antifungals. Therefore, at present, the mycological diagnosis is correctly based on laboratory evidence, but the antifungal treatment is undergoing a growing tendency to revert back to being empirical, as it was in the last century. Additionally, reports of clinical resistance to antifungals are steadily increasing, leaving mycologists with the dilemma of having higher rates of clinical resistance and fewer isolates to study. One of the explored options to circumvent these problems is to couple non-cultured based diagnostics with molecular-based detection of intrinsically resistant organisms and the identification of molecular mechanisms of resistance (secondary resistance). This idea would also overcome one of the main drawbacks of conventional AST techniques, the time needed to get confident results (>24 h) [5,6,7,8].

The aim of this work is to review the available molecular tools for antifungal resistance detection, their limitations, advantages, and development gaps.

## 2. Intrinsic Resistance Detection

As with other microorganisms, antifungal resistance is a broad concept that can be divided into clinical and microbiological resistance. The former was defined as the lack of inhibition of a microorganism in the infection site and it is related to different factors dependent on the drug, the patient, or both, rather than with the microorganism that causes the infection [9,10]. On the other hand, microbiological resistance depends on the particular characteristics of the microorganism and it can be subdivided into primary or intrinsic and secondary or acquired resistance. The results obtained in the AST give an idea of both microbiological resistance types.

Intrinsic microbiological resistance is the innate ability of a fungal species to resist the activity of a particular antifungal drug due to its inborn functional or structural features (e.g., absence of the drug target, inaccessibility of the drug into the cell). This resistance is exhibited by all strains of the same species of a fungus and is not related to exposure to the antifungal. On the other hand, secondary microbiological resistance is developed after antifungal treatment (in vivo or after environmental exposure) and is observed in particular strains of a normally susceptible species. These resistance phenotypes are due to genetic alterations that are manifested in a stable or in a transitory way [11,12]. Intrinsic and secondary resistance usually share the same molecular mechanisms. As an example, we can state the intrinsic fluconazole (FLC) resistance in *Aspergillus fumigatus* due to the naturally occurring T301I substitution at its Cyp51Ap [13] and the secondary mechanism of FLC resistance in *Candida albicans* attributable to an equivalent substitution (T315A) at its Erg11p [14].

Intrinsic resistance was defined by CLSI as “inherent or innate (not acquired) antimicrobial resistance which is reflected in wild-type antimicrobial patterns of all or almost all representatives of a species. Intrinsic resistance is so common that susceptibility testing is unnecessary” [15]. EUCAST lists a species as intrinsically resistant to an agent when “all or a vast majority of their strains exhibit minimal inhibitory concentration (MIC) values that are so high that the agent should not be considered for either therapy or clinical susceptibility testing” [16]. These definitions coincide in that AST is not necessary to be performed since all the strains of the species are resistant to that particular drug. Thus, in some microorganisms/drug combinations, taxonomy would act as an AST subrogate marker. On this topic, the CLSI already included in M60 document the sentence “Isolates of *Candida krusei* are assumed to be intrinsically resistant to FLC, so their MICs should not be interpreted using this scale.” (referring with scale to susceptible, susceptible dose-dependent, and resistant) [17]. Moreover, other species as *Aspergillus fumigatus* or other groups of fungi (Basidiomycetes and Mucorales) are being evaluated to be considered intrinsically resistant to FLC and to echinocandins, respectively.

There are powerful tools able to accurately identify intrinsically resistant fungal species from culture as Matrix-assisted laser desorption ionization-time of flight (MALDI-TOF) [18,19,20,21]. However, the identification of filamentous fungi is often compromised by difficulties in the extraction steps and by the fact that some cryptic resistant species are not yet included in the databases. The first problem could be resolved by performing extended extraction procedures [22] or using special culture media [23]. Despite the usefulness of MALDI-TOF as a technique, molecular-based identification (DNA sequencing) is still the gold-standard for fungal taxonomy [24].

Since the beginning, DNA-based identification methods in medical mycology faced a huge problem: choosing a gene (or a portion of a gene or genome region) useful in a clinical laboratory. To fulfill this objective, this hypothetical gen would have the following characteristics: it has to (i) have high inter-species and low intra-species variability, (ii) have a short sequence with high discriminatory power, (iii) be unique for all fungal species, (iv) make use of universal primers (same pair of primers for all species), and (v) be easily amplified [24]. These five points were condensed on the DNA barcoding concept that was aimed to allow accurate and fast species identification [25,26,27,28,29,30]. However, the truthfulness of these molecular-based identification procedures depends on the correct previous taxonomic classification of the used control strain [31]. This fact leads us to a paradox where molecular taxonomy’s objective is to improve classical taxonomy, but the former needs the last to achieve this goal.

There are several reports published regarding which gene to choose. However, any of the described molecular markers fulfill all the described criteria. The one that came closest to be the standard for fungi is the gene region known as ribosomal DNA internal transcribed spacers regions (ITS) [26,32,33]. This region is being successfully used for *Candida* spp. identification. Several reports used ITS-based PCRs to rapidly identify *Candida* spp. at the species level, including intrinsically resistant (e.g., *C. krusei*/FLC) [34] and less susceptible cryptic species [35,36,37,38]. Similarly, for Mucorales, ITS alone is a correct DNA marker with enough discriminative power to identify the currently accepted morphospecies of *Mucor*, *Lichtheimia,* and *Rhizopus* [39,40]. For other genera, ITS sequencing or ITS-based PCR identification is not enough or is not the correct method. For *Trichosporon* spp., intergenic spacer regions (IGS1) (and not ITS) sequencing unambiguously identify all *Trichosporon* species [41], while for most pathogenic filamentous fungi, a multilocus DNA-barcoding approach is needed [42,43]. *Aspergillus* spp. identification to sections/complexes level is based on sequencing of ITS regions [44,45] but a secondary marker is needed to allow the identification at the species level. For this genus, calmodulin (CaM), Beta-tubulin (BenA), and the second-largest subunit of the RNA polymerase II (RPB2) were proposed as taxonomy secondary markers. The last is quite difficult to amplify in certain species. For BenA, a different number of introns and paralogous genes were described making the amplification with one set of primers difficult. On the other hand, CaM can be amplified easily with the same primer set in most of the species making this gene the proposed secondary marker for *Aspergillus* spp. identification [46,47,48]. For *Fusarium* species, its identification is based on the sequencing of the ITS regions plus RBP2 and a portion of the translation elongation factor 1 alpha (TEF1) [49].

The described knowledge allowed the development of several techniques able to identify intrinsically resistant and cryptic (less susceptible) species. Most of these techniques and procedures designed to identify fungal pathogens were developed in research laboratories (in-house PCRs) and were barely used in clinical settings. On the other hand, there are few commercially available molecular-based methods in clinical use.

### 2.1. Intrinsic Resistance Detection by Commercially Available Molecular Taxonomy-Based Method

There are few FDA-cleared molecular-based methods capable of identifying fungal pathogens [50]. Some of them are able to identify them directly from positive blood culture bottles (e.g., FilmArray, Biofire–Biomerieux; *Candida* PNA FISH assay, OpGen; T2*Candida*-Biosystems and SeptiFast-Roche) [51] or from other clinical samples [52]. The major common limitation of these methods is the narrow coverage for fungal pathogens and the low impact in the selection of specific antifungal treatment [4,51]. The newest version of Biofire Filmarray is able to detect *C. albicans*, *C. parapsilosis sensu stricto*, *C. glabrata sensu stricto*, *C. tropicalis*, *C. krusei* (intrinsic FLC-resistant), *Cryptococcus neofromans/gattii* complex (intrinsic echinocandin resistant), and *C. auris* (multidrug-resistant). This last species showed a high prevalence of FLC resistance (>90% of the strains are considered FLC resistant). Despite that almost all *C. auris* strains show this phenotype, it was demonstrated that this resistance is acquired [53]. T2*Candida* panel (Magnetic resonance-based from T2 Biosystems) and LightCycler^®^ SeptiFast MGRADE system (Real-Time PCR from Roche Diagnostics) are able to rapidly (<5 h) diagnose candidemia utilizing whole blood directly from patients with a great level of detection (1CFU/mL for T2*Candida*) [54,55], a good specificity (>95% and >72%, respectively) and sensitivity (>60% and >90%, respectively) in real-life clinical settings [56,57]. However, both methods are able to identify only the most commonly isolated *Candida* spp. (*C. albicans*, *C. tropicalis*, *C. parapsilosis*, *C. krusei*, and *C. glabrata*) and *Aspergillus fumigatus sensu stricto* (Septifast only) [52,57]. The described diagnostic capability allows the clinicians to rapidly start an antifungal therapy helping with antifungal stewardship but it gives false-negative results when none of the named *Candida* spp. (or *A. fumigatus*) are the etiological agent of the infection (a huge problem especially given the shift in *Candida* spp. epidemiology) [52]. Moreover, considering that the current first-line treatment for candidemia are echinocandins, the identification to species level of these five common *Candida* spp. has little impact on the choice of antifungal treatment since none are intrinsically resistant to this class of antifungals (with the exception of Biofire that can detect *Cryptococcus* spp.). However, the Infectious Diseases Society of America (IDSA)guidelines propose echinocandins as the first treatment option for deep *Candida* infections (including candidemias) caused by any species of this genus. [58]. This guideline only suggests to study the echinocandin susceptibility of the strains isolated from patients who had a prior echinocandin treatment and in those patients with *C. glabrata* or *C. parapsilosis* infections. In this case, none of the described methods (filmarrays, T2, Septicheck, etc) is helpful since no strain is available to perform the required AST (as they are non-culture based methods). On the other hand, both methods are able to identify the intrinsically FLC-resistant *C. krusei* (Biofire can identify *C. auris* also) and *C. glabrata* (higher FLC doses as treatment).

Another important development in fungal diagnostics is the real-time PCR kit, commercialized by Pathonostics, designed to diagnose aspergillosis and to detect markers of secondary azole resistance named AsperGenius^®^. The tests are divided into two panels, one able to identify the etiological agent and the other to detect azole resistance. The first panel identifies aspergillosis caused by *A. fumigatus*, *Aspergillus terreus,* and *Aspergillus* spp. [59,60]. This test was evaluated clinically with good results in serum (sensitivity > 78% and specificity of 100% for species with a limit of detection >10 genomic units of *A. fumigatus*) [60], plasma (80% sensitivity and >77% specificity) [61], and in broncoalveolar lavage (BAL) (>80% sensitivity and >90% specificity for hematological and ICU patients) [62,63]. The biggest advantage of this diagnostic kit is its ability to detect the intrinsic amphotericin B resistant *A. terreus* and the fact that it has no false-negative results for aspergillosis caused by non-*A. fumigatus* and non-*A. terreus* (other available kits are able to detect only *A. fumigatus*) [64]. Moreover, and as an unexpected result since the kit was not designed to do so, AsperGenius^®^ can detect intrinsically azole and amphotericin B resistant cryptic species of the *Aspergillus* section Fumigati as *A. lentulus* and *A. felis*. These results were obtained by using the AsperGenius^®^ resistance panel where the TR_34_ target (see details in the next section) was negative for these two cryptic species and the melting curves for the *CYP51A* mutations were also different [65].

**Figure 1:**
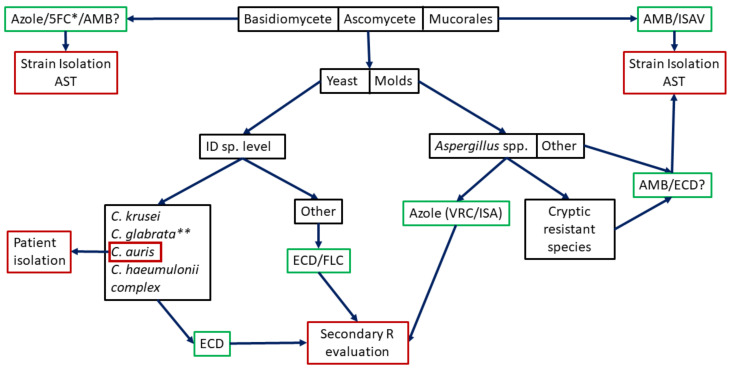
Potential algorithm for intrinsic resistance studies. Black boxes indicate where potential molecular methods would be used (most not designed yet). Green boxes indicate used empirical treatments. Red boxes indicate needed actions (patient isolation if *C. auris* is identified, the need of secondary resistance evaluation or the strain isolation and AST). 5FC: 5-fluorocytosine, AMB: amphotericin B, ISAV: isavuconazole, ID: Identification, ECD: echinocandins, FLC: fluconazole, VRC: voriconazole. * Combined with AMB for *Cryptococcus* spp. ** FLC Susceptible Dose-Dependent/Resistant.

After this short summary of the main commercially available molecular-based method able to detect intrinsically resistant fungi, it is clear that a bigger effort is needed to include more species to the existing panels or to design more comprehensive ones. Efforts should be focused on the differentiation of the fungal groups of species with known intrinsic resistance to certain antifungals directly from clinical samples. The main groups of fungi to be differentiated in order to select a correct antifungal treatment and to have a real impact on mortality [66] are: (i) ascomycetous from basidiomycetous yeasts, (ii) agents of hialohifomycoses from Mucorales, and (iii) *Candida* spp. from filamentous fungi since the last of each of these pairs of pathogens are intrinsically resistant to echinocandins, voriconazole, and FLC, respectively (Figure 1). One promising tool able to fulfill at least in part these requirement seems to be the PCR-electrospray ionization mass spectrometry that is being tested for mycoses diagnostics [67,68,69,70,71].

### 2.2. Intrinsic Resistance Detection by in-House Molecular-Based Method

Several reports of in-house PCRs demonstrated the feasibility and the practical potential of the different methods to uncover intrinsic resistant or less susceptible fungal species, both starting from colonies and from clinical samples. Within the in-house methods developed, the ones that stand out are able to detect cryptic *Candida* spp. that showed reduced susceptibilities to different antifungal agents as the *C. glabrata*, *C. parapsilosis,* and *C. albicans* species complexes. Different formats were used including a multiplex PCR able to detect all nine species (*C. glabrata sensu stricto*, *C. nivariensis*, *C. bracarensis*, *C. parapsilosis sensu stricto*, *C. orthopsilosis*, *C. metapsilosis*, *C. albicans*, *C. dubliniensis,* and *C. africana*) [72], several multiplex PCRs for the detection of each species of one of the complexes [38,73,74], PCRs coupled to restriction enzyme digestions [75,76], high resolution melting curves [77], a multiplex real-time PCR using molecular beacons [75], etc. Some of these methods were successfully used to evaluate the prevalence of these cryptic species in strain collections [36,78,79,80]. In response to the need for molecular tools to identify *C. auris* due to its high FLC resistance rate (>90%), a classical and a real-time PCRs based on ITS amplification were published. The first included a one single tube PCR able to uncover *C. auris* and *C. haeumulonii* with an internal reaction control [81] and a specific PCR for *C. auris* DNA amplification [82]. The published real-time PCRs are capable to detect *C. auris* alone or *C. auris*, *C. haeumulonii*, *C. duobushaemulonii,* and *C. lusitaniae* by analyzing melting curves [82].

Very recently, qPCRs (Sybrgreen and with TaqMan probes) targeting the *C. auris* ITS2 region were designed in order to detect *C. auris* DNA from skin and surveillance samples (limits of detection of 4 and 1 *C. auris* CFU/PCR, respectively) [83,84,85]. Moreover, these qPCRs can be coupled with a second panel of primers to uncover markers of secondary resistance as *ERG11* and *FKS1* mutations responsible for FLC and echinocandin resistance, respectively [86]. These *C. auris* detection tools are examples of the ideality of molecular diagnostics of antifungal resistance using clinically important samples. They showed the potential, sensitivity, and usefulness of these tools. However, as in many other molecular tools, a suspicion of the presence in a sample of a particular species is needed in order to select the molecular method to detect them. Thus, these tools are useful in confirming an infection or colonization caused by the suspected agent and have a great negative predictive value.

For Mucorales, most of the in-house methods are designed to be used directly from clinical samples since it is difficult to isolate these fungi from the culture [87,88]. The usefulness of these molecular methods is that a positive result is the needed evidence to start an antifungal treatment with amphotericin B. These methods are mainly based on the amplification and subsequent sequencing of rDNA using total DNA isolated from formalin-fixed and fresh tissues [89,90,91,92,93]. Moreover, there were reports of using different molecular targets as a semi-nested PCR able to identify several Mucorales species [94], an RFLP-PCR used in biopsies [22,95], a pan-Mucorales PCR based on the amplification of the spore coating protein (*CotH*) [96], a qPCR able to detect *Rhizomucor* spp., *Mucor* spp. *Rhizopus* spp. and *Lichtheimia* spp. in broncoalveolar lavage samples [97], a qPCR based on ITS amplification [98], etc.

### 2.3. Is Species Identification Enough as Surrogate Marker of Intrinsic Resistance or Should We Go Further?

There are some examples of fungal species that were divided into clades, varieties, or types that showed differences in their antifungal susceptibility. One of the first noted examples was the differences in 5-fluorocytosine (5FC) susceptibilities of different *C. albicans* clades (resistance to 5FC seems restricted to clade I) [99]. Other clinically relevant examples to be cited are *Cryptococcus neoformans*/*gattii* complex species, *C. auris,* and *Trichophyton mentagrophytes*. The named basidiomyceteous yeasts are divided into genetic varieties that have different epidemiological cut-off values for multiple antifungal agents [17,100,101]. *C. auris* was firstly considered intrinsically resistant to FLC (MIC > 64 µg/mL) but after studying a more geographically diverse collection of strains it was established that this phenotype was shown by strains of the clade I, III, and IV, while most of the strains of the clade II showed lower FLC MICs [102,103]. Similarly, *T. mentagrophytes* was divided into different genetic types. Type VIII isolates (from India) showed a high level of terbinafine resistance [104].

Looking at this data, we can assume that the identification of clades, types, and varieties of particular species showing reduced antifungal susceptibility would be a useful surrogate marker of resistance. However, to do so, continuous antifungal surveillance and molecular epidemiology studies are needed to increase the number of species to be included in this list.

## 3. Secondary Resistance Detection

The number of described secondary antifungal resistance mechanisms differs between drugs. For amphotericin B, there are few reports on secondary antifungal resistance. It is so rare that it has raised questions about the ability of AST methods to detect it [105,106]. These secondary resistance mechanisms were described 40 years ago or were barely studied [107,108]. For azole drugs, there is a wide range of different mechanisms [10] while for echinocandins, secondary clinical resistance seems mainly linked to amino acid substitutions at its target (Fksp) [10,11]. It should be also stated that clinically important secondary resistance is related to genetically stable mutants selected during treatment in a multistep process. This process involves a cell stress step produced by the drug, followed by an adaptation step that ends up in a stable escape mutant. These adaptation steps include the overexpression of genes that compensate for the drug-produced alterations, overexpression of stress response pathways, chromosome rearrangements, etc. These processes are usually reversible if the drug pressure is reduced or is eliminated. On the other hand, stable escape mutants retain the phenotypic traits that classified the strain as resistant (MICs surpassing the clinical breakpoint) whether the drug pressure is maintained or not [109]. These last kinds of mutants are the ones that shall be detected by molecular tools to confirm a resistant phenotype and in some cases, its detection is considered an independent risk factor for treatment failure [110,111].

**Figure 2:**
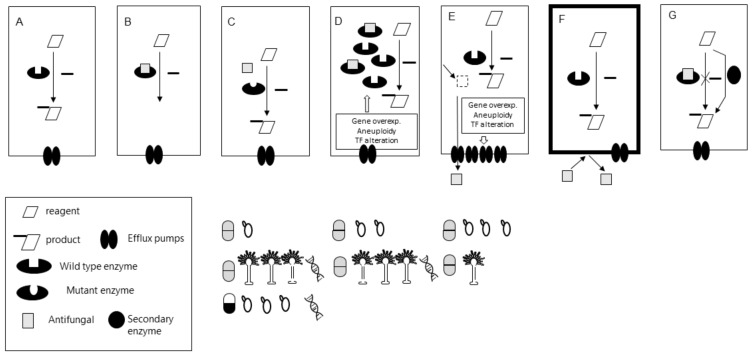
(**A**) Essential enzymatic reaction for a fungal cell. (**B**) Inhibition of the reaction by the antifungal drug. (**C**,**D**) Alterations of the interaction drug-enzyme. (**C**) Mutation on the drug target (less efficient or non-interaction). (**D**) Overexpression of the drug-target. In the box are the most commonly described mechanisms leading to overexpression. (**E**,**F**) Reduction of the cytoplasmic drug concentration. (**E**) Overexpression of efflux pumps (e.g., CDRs) by different underlying mechanisms (described in the box). (**F**) Impermeability. The drug is not trespassing the membrane (e.g., no transporter available as happens with 5-fluorocytosine and *Histoplasma capsulatum*. (**G**) Metabolic bypass. Grey and black and White pill symbols represent azole and echinocandin antifungals resistance mechanisms, respectively. Yeast and *Aspergillus* graphics under each of the mechanisms represent the description of each particular mechanism in *Candida* spp. and *Aspergillus* spp. respectively. The number of Yeasts and *Aspergillus* graphs show the relative prevalence of each of the mechanisms in *Candida* and *Aspergillus* spp. clinical strains, respectively. DNA graphs represent molecular tools’ availability to detect that particular mechanism in a drug/fungi combination.

The ways in which fungi acquire the ability to escape the action of antifungals can be divided into three groups of mechanisms: (i) alteration of drug-target interaction (target modification and target hyper-production), (ii) reduction of the cytoplasmic concentration of the drug (overexpression of efflux pumps and reduction of drug penetration), and (iii) metabolic by-pass (Figure 2). The prevalence of each of these mechanisms depends on the drug/fungi combination. Briefly, for azole agents, overexpression of efflux pumps followed by mutations at the azole drug target (*ERG11*) and the overexpression of *ERG11* are the main resistance mechanisms in *Candida* spp. On the other hand, *CYP51A* substitutions followed by *CYP51A* overexpression coupled with *CYP51A* substitutions are the main mechanisms of azole resistance in *Aspergillus* spp. Conversely, *Cryptococcus* spp. azole resistance mechanisms seem to be related mainly with *ERG11* mutations and chromosome rearrangements that lead to the overexpression of *ERG11* and transcription factors genes that increase the expression of efflux pumps. Turning to echinocandins, target modification (*FKS* mutations) is the most important mechanism of echinocandin resistance despite the studied fungal species. For a more detailed description of molecular mechanisms of antifungal resistance, interested readers are referred to the following references [11,72,112,113,114,115,116,117,118].

### 3.1. The Bottlenecks of Secondary Resistance Molecular Detection

For intrinsic resistance detection, a wide range of taxonomy markers are available. As mentioned, one of the most used is the ITS region which is a multicopy universal region of the fungal genome. These characteristics imply that one pair of primers can be used to detect multiple fungal species with high sensitivity (each fungal cell can carry between >10 to >1000 copies of this DNA region) [119]. Oppositely, for the case of resistance markers, there are several bottlenecks in terms of sensitivity and methodological complexity. The first is obviously the low sensitivity since the genes to be evaluated are a single copy (two in diploid organisms). The second is that we should uncover mutations in a gen rather than detecting the presence of a gen. The third is that different mechanisms involving different genes and/or mutations are responsible for similar or equal phenotypes. The fourth is that in some cases we must evaluate overexpression and not just the presence and/or existence of mutations. Fifth, in the case of diploid organisms, there are mechanisms that are dominant (a mutant allele gives the phenotype) limiting the use of classic PCR. Sixth, there are no universal primers (each species uses different oligonucleotides), so we must identify the etiological agent before detecting resistance mechanisms. The seventh is that some phenotypes are the result of a combination of mechanisms. The eighth is that we can only detect known mechanisms, as opposed to phenotypic or whole-cell methods such as MIC assessment that detect all mechanisms.

### 3.2. Available Molecular Tools. Which Secondary Mechanisms Are We Able to Detect?

Having in mind the described bottlenecks, in-house and commercially available tools were designed to detect the mechanisms regarded as the unique responsibilities of particular resistance phenotypes. Thus, most of the published tools are able to detect some of the *CYP51A* and *FKS* mutations linked with triazole and echinocandin resistance in *Aspergillus fumigatus* and *Candida* spp., respectively.

There are no standard methods to detect alterations on gene coding antifungal targets. However, several methods were published to be used mostly from isolates. The first reports simply used PCR amplification followed by sequencing [120,121,122,123,124,125]. Later specific PCR-based methods designed for the detection of particular mutations were published using different methods and formats (Table 1 and Table 2).

#### 3.2.1. Triazole Secondary Resistance in *Aspergillus* spp.

**Figure 3:**
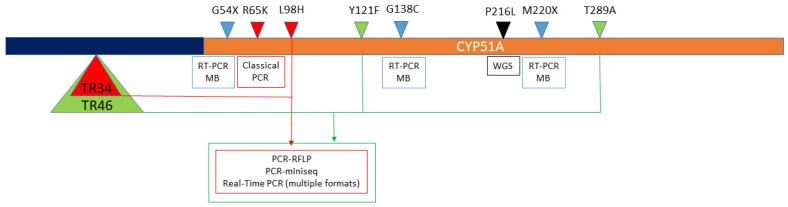
Mutations in A. fumigatus cyp51A (brown bar) and its promoter (5´UTR) region (dark blue bar) detected by the available molecular methods. Boxes indicate the molecular methods used to detect the mechanisms that are over them. Arrow heads represent the mutations linked with triazole resistance. Green and red arrow heads show the combined mechanisms that include CYP51A mutations together with promoter alterations described as tandem repetitions (TR). Light blue arrow and black heads represent single-nucleotide mutations detected by RT-PCR coupled with molecular beacons (RT-PCR MB) and whole-genome sequencing (WGS).

The development of molecular tools for the detection of triazole resistance mechanisms in *A. fumigatus* was less complicated than for other fungal species since fewer mechanisms were described. Resistance linked to mutations at *CYP51A* has been detected by in-house classical PCRs (followed by sequencing, digestion, minisequencing-SnaPshot) [131,132,133,134,135], astringent classical PCRs [139], real-time PCRs (coupled with taqman probes, molecular beacons probes, locked primers, sybrgreen followed by melting curves analysis and FRET probes) [126,127,128,129,130,136], loop-mediated isothermal amplification (LAMP) [138], whole-genome sequencing (WGS) [140], etc. The majority of these methods are able to detect the most common mechanisms as the promoter alterations (TR34-L98H and TR46-Y121F) (Table 1). The resistance mechanisms that include promoter alterations are also detected by the two commercially available methods (not FDA approved yet) named AsperGenius [61,62,63,141] and MycoGENIE [142]. Both diagnostic kits share the format (multiplex real-time PCR) and the capacity of detection of TR34-L98H mutations. As described earlier, AsperGenius is able to detect DNA of intrinsically resistant species while MycoGENIE is able to detect only wild-type and resistant *A. fumigatus sensu stricto* isolates. AsperGenius also covers the second most common mechanism of triazole resistance (TR46-Y121F-T289A). One of the few molecular-based methods capable to detect other mutations (G54, M220, and G138C) is an in-house real-time PCR coupled with molecular beacons designed in a two-panel format. The first panel detects itraconazole or triazole cross-resistance while the second panel can differentiate G54W (ITC-PSC cross-resistance) from other substitutions at the residue 54 conferring ITC resistance. Moreover, in the same panel, molecular beacons that confirm resistance mechanisms were included [126]. For less prevalent mechanisms, quantitative real time PCRs and PCR followed by minisequencing were reported as feasible tools to detect the overexpression of efflux pumps and the uncommon mutations at *CYP51B*, respectively [129,144] (Figure 3).

Another point to consider is that five out of 15 (33%) described methods were tested using clinical samples (sputum, BAL, Formalin-fixed, and paraffin-embedded tissues). Two of them are commercially available methods (see Table 1).

#### 3.2.2. Azole Resistance in *Candida* spp.

Turning to *Candida* spp., the panorama is different. Molecular mechanisms of azole resistance in *Candida* spp. are relatively well studied. However, its detection is complicated since each species can have different mechanisms and because an individual mechanism is not always strictly correlated with strain´s MIC. Firstly, some Erg11p amino acid substitutions in *Candida* spp. were described but not validated as exclusive causatives of azole resistance [125,153]. Secondly, in the majority of the cases, the resistance phenotype is due to a combination of mechanisms. They can include two or more of the following: (i) mutations at different genes of the ergosterol biosynthesis pathway (especially in *ERG11* but also in *ERG3*) [125,153,154], (ii) overexpression of *ERG11* due to the gain of function mutations in transcription factors or due to aneuploidies (complete or partial chromosome duplications) [155,156], overexpression of efflux pumps [157], etc. (Figure 1). Additionally, it was demonstrated that aneuploidies can lead to tolerance or resistance to multiple unrelated drugs [158]. This multiplicity of mechanisms makes the use of a multiplex format mandatory for the molecular detection of resistance to azoles in *Candida* spp., meaning, the sequencing of several genes coupled with the expression evaluation of others.

Some of the latest proposed approaches are the use of next-generation sequencing (NSG) and whole-genome sequencing (WGS) to detect several mutations in different genes of the ergosterol biosynthesis pathway (*ERG11* and *ERG3*) and transcription factors that regulate efflux pump expression (e.g., *TAC1* and *PDR1*) together with *FKS* mutations [102,149]. These techniques are able to detect novel DNA alterations but they are limited to reference labs due to their high cost (equipment, reagents, and the complexity of data analysis) [149]. In the few published reports that used NGS, strain collection was studied, but standardized AST was firstly used as a screening tool. Thus, NGS was used to uncover the mechanism responsible for the already known resistance phenotype and not as a diagnostic tool of antifungal resistance [102,150].

The most important exception to what was stated is the detection of the FLC resistance mechanism in *C. auris*. Unlike other *Candida* spp., FLC resistance in *C. auris* was linked only to a limited number of Erg11p substitutions (mostly Y132F, K143R, F126T, I466M, and Y501H). The prevalence of these mutations is different in each of the described 4 geographical clades of *C. auris* and some substitutions are almost exclusively found in one clade (e.g., I446M in clade IV–South American) [102,151]. This fact makes feasible the detection of *C. auris* resistant strains by molecular tools. Xin Hou et al. reported an asymmetric real-time PCR coupled with molecular beacons able to identify Y132F and K143R Erg11p substitutions together with an *FKS* mutation (S639F) responsible for echinocandin resistance [152].

#### 3.2.3. Echinocandin Resistance in *Candida* spp.

As for *C. auris*, the molecular detection of echinocandin resistance in *Candida* spp. can be performed using relatively simple methods. This detection is aided by the fact that resistance to echinocandins has two fundamental characteristics: it is almost exclusively linked to a limited number of mutations [11,159] and its presence is an independent risk factor for echinocandin therapy failure [111]. Although echinocandin resistance was described in several *Candida* spp., its prevalence is higher in *C. glabrata* and in *C. albicans*. This fact is reflected in the published technological developments. The reported molecular tools for the detection of *C. glabrata* and *C. albicans FKS* mutants include classical PCRs [36,144,147], Sanger [150], and next-generation sequencing for both species [149]. There are methods designed exclusively for the detection of *C. albicans FKS* mutants as a real-time PCR coupled with molecular beacons [148]. On the other hand, asymmetric real-time PCR coupled with molecular beacons with melting curve analysis [143], Luminex Mag-Pix assay [146], and whole-genome sequencing were used to detect *C. glabrata FKS* mutants [145]. More details on the methodologies including their limitations are described in Table 2.

Most of the described tools detect the most prevalent mutations that are responsible for the most pronounced phenotype (e.g., at the residues S629 and S663 in *C. glabrata* Fks1p and Fks2p). On the other hand, the two reported classical PCRs are also able to detect less common mutations that showed lower MIC values as the substitutions at the residues D648, P649, and R1361 at *C. albicans* Fks1p and D632 at *C. glabrata* Fks1p. However, these methods showed intrinsic limitations of classical PCRs as their inability to detect heterozygous mutants [36,144,147]. All PCR-based methods (classical and real-time) have common problems with the design of primers and allele-specific probes. The first is the presence of synonymous or silent polymorphisms at *C. albicans* and *C. glabrata FKS* hot spots that were partially fixed by the introduction of a wobble base in the probe (or degenerated probe) [148] while others designed multiple primer combinations trying to avoid the residues where polymorphisms were reported [144,147].

## 4. Conclusions

The clinical predictive value (or lack thereof) of the uncovering of a molecular resistance mechanism may be relative. The “90–60 rule” applies to both molecular and whole-cell antifungal susceptibility evaluation. This “rule” roughly states that ~90% of infections due to susceptible isolates respond to the correct antifungal treatment, whereas ~60% of the infections caused by resistant isolates (or infections treated with an incorrect drug) respond to therapy [160].

Molecular methods can be used to detect intrinsic and secondary resistance. There are in-house and commercially available methods. The major common limitation of these methods is the narrow coverage for fungal pathogens and the low impact in the selection of specific antifungal treatments.

The commercially available diagnostic tools have a low impact on the selection of specific antifungal treatments. A bigger effort is needed to include more species in the existing panels. Efforts should focus on the differentiation of the fungal groups of species with known intrinsic resistance to certain antifungals directly from clinical samples.

The rDNA (ITS) is a good marker for intrinsic resistance detection for Mucorales and for *Candida* spp. On the other hand, for most pathogenic filamentous fungi, a multilocus DNA-barcoding approach is needed.

As in any other molecular tools, a suspicion of the presence of a particular species in a sample is needed in order to select a particular molecular method. Thus, these tools are useful in confirming an infection or colonization and have a great negative predictive value.

The identification of clades, types, and varieties of particular species showing reduced antifungal susceptibility would be a useful surrogate marker of resistance.

There are more bottlenecks in terms of sensitivity and methodological complexity for the detection of secondary than for intrinsic resistance by molecular methods. These limitations include single copy vs. multiple copy genes, mutation detection and or expression evaluation vs. presence of a gene, several mechanisms with the same phenotype vs. one gene same diagnosis, no universal primers vs. universal primers, etc.

Molecular methods can only detect known mechanisms, as opposed to phenotypic or whole-cell methods such as MIC assessment that detect all mechanisms.

There are no molecular methods able to detect amphotericin B (AMB) resistance.

It is difficult to detect azole resistance in *Candida* spp. due to the multiplicity of mechanisms involved. NSG and WGS were used in reference labs to confirm and study already known resistance phenotypes.

Most of the published secondary resistance detection tools are able to detect some of the *CYP51A* and *FKS* mutations linked with triazole and echinocandin resistance in *Aspergillus fumigatus* and *Candida* spp. (specially *C. albicans*, *C. glabrata*, and *C. auris*), respectively

*A. fumigatus* triazole resistance molecular diagnosis is mostly limited to the detection of *CYP51A* promoter alterations (TR34 and TR46).

There are multiple technological options to detect echinocandin resistance mechanisms in *C. albicans* and *C. glabrata*.

There are clinical settings where resistance mechanism detection would be valuable as places where triazole resistance in *A. fumigatus* prevalence surpass 10%, high use of empirical treatment, etc.

## Figures and Tables

**Table 1:** Molecular tools for the detection of mechanisms of triazole resistance in *Aspergillus* spp.

Format	Target and Technique Characteristics	Samples	Detected Mechanism	Points to Consider	Reference/Publication Year
Real-time PCR with molecular beacons	Two panels of multiplex PCRs. The first detects ITC and cross azole resistance. The second detects PSC resistance and confirms the coexistence of TR and L98H.	Isolates strains	G54X (ITC-R), G54W (ITC/PSC-R), M220X (ITC-R), G138X/C * (Cross-R), TR34-L98H (Cross-R)	60 strains (52 clinical and 8 lab mutants) harboring G54X, M220X, G138C, TR34-L98H, TR34 alone, and L98H alone.	[126] 2008
Real-time PCR using Taq-man probes	CYP51A ORF and promoter	Formalin-fixed and paraffin-embedded tissue	TR34-L98H	Only one patient	[127]/2010
Nested 2 step PCR. Firstly classical PCR in 2 tubes (outer). Second PCR in real-time format with molecular beacons	CYP51A ORF (first classical PCR) and promoter plus a partial ORF amplification (second classical PCR). Molecular beacons bind to secondly amplified targets.	Sputum and BAL	TR34-L98H G54, G138, and M220.	DNA extraction using the MycXtra fungal DNA extraction kit (Myconostica Ltd.). 22 samples 5 proven and 17 probable aspergilloses.	[128]/2011
Real-time PCR–FRET probes with melting curves analysis	CYP51A ORF and promoter.	Isolated strains (clinical)	TR34-L98H G54, G138, and M220.	215 [129] and 103 [130] *A. fumigatus sensu stricto* included. TR34-L98H (*n* = 4) was the only detected mechanism. There were no G54, G138, and M220 mutations (only wild type confirmation)	[129,130]/2010 and 2012
Classical PCR and nested PCR followed by sequencing.	3 individual PCRs able to amplify CYP51A promoter and fractions of its ORF	Isolated strains [131] and clinical samples (BAL and tissue) [132]	TR34-L98H and M220	Developed using strains and clinical samples [131] and tested in a clinical setting [132].	[131,132] 2012/2014
PCR-RFLP (amplification followed by AluI digestion)	A promoter and a CYP51A ORF fragment (289 bp) [133]. Later, a bigger fragment was used [134].	Isolated strains (clinical and environmental)	TR34-L98H [133] and TR34-L98H and TR46-Y121F-T289A [134]	Good correlation with MIC but false negative (isolates harboring other mechanisms)	[128,130] 2014 and 2017
Single tube PCR followed by minisequencing	A multiplex classical PCR followed by purification and detection of 21 SNPs at *CYP51A* and *CYP51B* by single-base extension reaction (SNaPshot^TM^) using a Sanger-based sequencer.	Isolated strains	TR34, G54, L98, G138, M220, S297, G448, and 12 CYP51A polymorphisms. Two CYP51B polymorphisms were also included.	79 clinical and 21 environmental isolates. No resistant mutants but several with *CYP51A* and *CYP51B* polymorphism.	[135] 2015
Real-time PCR with locked nucleotide probes	Partial CYP51A ORF amplification. Detection of both wild type and mutant (L98H and Y121F) alleles.	Isolated strains	TR34-L98H and TR46-Y121F-T289A. It only detects the mutation in the ORF, not the promoter alteration	Detection of 6 L98H mutants/166. No Y121F mutants were detected.	[136] 2016
Quantitative real-time PCR with sybrgreen	Different efflux pump genes.	10 clinical strains with high azole MICs.	Detection of overexpression of efflux pumps	80% of the strains showed > 5 –fold increase of *cdr1B* gene expression.	[137]/2013
Loop-mediated isothermal amplification (LAMP)	TR34 promoter alteration	Clinical strains	Detection of TR34 alone.	Rapid (<25 min) High sensitivity (10 genomic copies).	[138]/2019
Classical PCR using astringent conditions	TR34-R65K-L98H	Clinical strains	Detection of R65K mutation and TR34.	Low-cost detection of a triazole-cross resistance (TR34) and pan azole resistance (R65K)	[139]/2020
Whole-genome sequencing	Complete genome	Isolates from sequential clinical samples from two patients (one Aspergilloma and one invasive aspergillosis)	P216L	Complex to perform in a clinical setting. Potential to uncover any mechanism after analysis	[140]/2014
AsperGenius multiplex real-time PCR assay.	Two panels. The first is a taxonomy panel (based on 28S rDNA). The second is A. fumigatus sensu stricto resistance detection (melting curve analysis).	Isolated strains (*n* = 131) [63] were used for validation. Clinical samples. BAL (*n* = 22) [63] (*n* = 201) [62] (*n* = 124) [61] (*n* = 100) [141] were used for clinical evaluation (most from proven and probable invasive aspergillosis).	Detects intrinsic resistant species (*A. terreus* and cryptic species of the Fumigatii section) and the following secondary resistance markers separately: TR34, L98H, Y121F, and T289A.	Some cross-reactivity (false-positive results) were obtained when *R. oryzae* (*R. arrhizus*) and *P. chrysogenum* DNA is present at high concentrations. During validation, high sensitivity and specificity were proved (both >80%) [62]. TR34-L98H was the most prevalent mechanism [61,62,141]	[61,62,63,141]/2017–2015-2016–2016
MycoGENIE multiplex real-time PCR assay.	Identification of *A. fumigatus sensu stricto* (based on rDNA sequence) and TR34-L98H detection	Clinical samples		Possible false positive when aspergillosis is caused by non-*Aspergillus fumigatus* species.	[142]/2017

ITC: itraconazole. PSC: posaconazole. –R: resistance. X: any amino acid. SNPs: Single nucleotide polymorphisms. * mutations other than G138C have never been described, cross azole-R phenotype is known for G138C. BAL: Bronco-alveolar lavage.

**Table 2:** Molecular tools for the detection of mechanisms of azole and echinocandin resistance in *Candida* spp.

Organism	Format	Target and Technique Characteristics	Samples	Detected Mechanism	Points to Consider	Reference/Publication Year
*Candida glabrata*	Asymmetric real-time PCR coupled with molecular beacons with melting curve analysis	*FKS1* and *FKS2* hot spot 1 regions.	Isolated strains	Echinocandin resistance. 4 amino acid substitutions at Fks1p (F625, S629, D632, and I634) and 2 at Fks2p (F659 and S663). Melting curve analysis can differentiate different nucleotide substitutions in the same position (8 at *FKS1* and 7 at *FKS2*).	Tested with a blinded panel of 188 strains. 100% concordance with sequencing.	[143]/2016
	Classical PCR with astringent conditions	*FKS1* and *FKS2* hot spot 1 regions.	Isolated strains	Echinocandin resistance. 3 amino acid substitutions at Fks1p (F625, S6229, and D632) and 2 at Fks2p (F659 and S663).	Tested with a blinded panel of 50 strains. Not able to detect F659del mutants [144]. It was tested later and showed a 99.25% concordance with MIC values. One strain was misclassified as resistant due to a silent mutation [145].	[36,144]/2014 and 2017
	High-throughput microsphere-based assay using the Luminex MagPix technology	*FKS1* hot spot 1 and hot spot 2 regions.	Strain collection	Echinocandin resistance. It potentially can detect all the *FKS* mutations.	Screen a collection of 1032 strains.	[146]/2014
*Candida albicans*	Classical PCR with astringent	*FKS1* hot spot 1 and hot spot 2 regions.	Isolated strains	Echinocandin resistance. It detects 8 different substitutions at 5 Fks1p residues. Four at hot spot 1 (F641, S645, D648, P649) and one at hot spot 2 (R1361)	96% sensitivity. It can detect all the homozygous mutants included. Heterozygous mutants give false susceptibility due to method-inherent limitations of the classical PCR.	[147]/2015
*Candida albicans*	Allele-specific real-time PCR molecular-beacon	*FKS1* hot spot 1	Laboratory mutants generated by CSF pressure.	Echinocandin resistance. It detects 4 substitutions at the residue S645.	It was the first published method. It gave the proof of concept that it is possible to detect *FKS* mutations. Currently outdated.	[148]/2006
*Candida albicans, Candida glabrata and Candida parapsilosis*	NGS	6 genes linked with antifungal resistance (*ERG11*, *ERG3*, *TAC1*, Cg*PDR1*, *FKS1,* and *FKS2*)	Isolated strains. For validation, resistant strains with known mechanisms. Then, clinical resistant strains.	Azole and echinocandin resistance. New mechanisms were uncovered including one gain-of-function and one loss-of-function Cg*PDR1* mutations responsible for azole resistance and hypersusceptibility, respectively.	It demonstrates that a mixed population (mutated and WT) would be isolated from a patient during caspofungin treatment. It gave the proof of concept that it is possible to use NGS for extensive assessment of mutations responsible for antifungal resistance	[149]/2015
*Candida albicans* and *Candida glabrata*	WGS and Sanger sequencing	Sanger sequencing of *FKS* hot spot regions and WGS for azole resistance markers	Isolated strains	*FKS*, *ERG11*, *ERG3*, *UPC2*, *MDR1*, *MRR1*, *TAC1*, *CDR1,* and *CDR2*.	Strains showing echinocandin and/or azole high MIC values were studied. Used as a research tool and not as a diagnostic tool.	[150]/2017
*Candida auris*	WGS		Isolated strains	*ERG11* mutations	Worldwide strains were divided into 4 clades and each clade showed differential FLC susceptibility (Clade II lower MIC- no *ERG11* mutations)	[102]/2017
*Candida auris*	Asymmetric real-time PCR coupled with molecular beacons with melting curve analysis	One *FKS1* and two *ERG11* mutations.	Isolated strains	Echinocandin and FLC resistance. It can detect the main mechanisms of azole resistance (Y132F and K143R in Erg11p) and echinocandin resistance (S639F in Fks1p) in this species.	Some strains belonging to clade IV (South America) would be identified as false FLC susceptible since the most prevalent mechanism of FLC resistance is the substitution I466M [151].	[152]/2019

CSF: caspofungin, FLC: fluconazole. NGS: Next-generation sequencing. WGS: Whole-genome sequencing.

## Data Availability

Not applicable.
